# The Effects of LPM570065, a Novel Triple Reuptake Inhibitor, on Extracellular Serotonin, Dopamine and Norepinephrine Levels in Rats

**DOI:** 10.1371/journal.pone.0091775

**Published:** 2014-03-10

**Authors:** Renyu Zhang, Xiang Li, Yanan Shi, Yufeng Shao, Kaoxiang Sun, Aiping Wang, Fengying Sun, Wanhui Liu, Di Wang, Jingji Jin, Youxin Li

**Affiliations:** 1 School of Life Sciences, Jilin University, Changchun, Jilin Province, China; 2 State Key Laboratory of Long-acting and Targeting Drug Delivery System, Luye Pharmaceutical Company Limited, Yantai, Shandong Province, China; 3 School of Pharmacy, Yantai University, Yantai, Shandong Province, China; National Hospital of Utano, Japan

## Abstract

Triple reuptake inhibitors (TRIs) are currently being developed as a new class of promising antidepressants that block serotonin (5-HT), dopamine (DA) and norepinephrine (NE) transporters, thereby increasing extracellular monoamine concentrations. The purpose of this study was to investigate the effects of LPM570065, a novel TRI and a desvenlafaxine prodrug, on extracellular 5-HT, DA and NE levels in the rat striatum after acute and chronic administration relative to desvenlafaxine, using High Performance Liquid Chromatography (HPLC) and microdialysis. Acute administration was performed by providing rodents with oral solutions (0.06 mmol·kg^−1^ p.o.), oral suspensions (0.06 mmol·kg^−1^ p.o.) and intravenous solutions (0.04 mmol·kg^−1^ i.v.) of LPM570065 and desvenlafaxine. Oral suspensions (0.06 mmol·kg^−1^·day^−1^) of the two drugs were also administered for a 14-day chronic period. HPLC analysis revealed that LPM570065 rapidly penetrated the rat striatum, converted into desvenlafaxine and exhibited larger total exposure compared with the administration of desvenlafaxine. Microdialysis revealed that acute and chronic administration of oral suspension of LPM570065 increased the 5-HT, DA and NE levels more than the relative administration of desvenlafaxine. Unlike desvenlafaxine, acute administration of an intravenous LPM570065 solution did not induce the undesirable 90% decrease in extracellular 5-HT levels. In contrast to the fully dose-dependent elevation of 5-HT induced by desvenlafaxine, the acute administration of LPM570065 showed a capped increase in extracellular 5-HT levels when combined with WAY-100635. Additionally, forced swim test demonstrated that acute and chronic administration of LPM570065 reduced the immobility time more than the relative administration of desvenlafaxine. These data suggest that LPM570065 may have greater efficacy and/or a more rapid onset of antidepressant action than desvenlafaxine and also counterbalance the harmful effects of desvenlafaxine on 5-HT neurotransmission related to 5-HT_1A_ autoreceptors. Thus, this new class of drugs, TRIs has the potential to provide a new therapeutic mechanism for treating depression.

## Introduction

Decades of effort have been expended trying to understand the pathophysiology of depression and to explore adequate pharmacotherapy of the disease. The typical mechanism and the majority of currently used antidepressants involve inhibiting serotonin (5-HT) and/or norepinephrine (NE) reuptake from the synapse. This treatment is consistent with the monoamine hypothesis of depression [1], which remains an important theory and continues to stimulate research. These pharmacological agents, including selective serotonin reuptake inhibitors (SSRIs) and serotonin-norepinephrine reuptake inhibitors (SNRIs), are often effective; however, the onset of therapeutic action is delayed by several weeks and approximately one-third of patients do not respond clinically to these agents [2], [3]. Moreover, many comorbid symptoms of depression, such as anhedonia, fatigue, motivation and cognitive impairment, are not effectively alleviated by these drugs [4], [5]. Indeed, these single- and dual-acting agents suffer from insufficient theory and demonstrate multiple limits in therapeutic efficacy.

In addition to these monoamines (5-HT and NE), earlier studies support diminished dopamine (DA) neurotransmission in the pathophysiology of depression. [6], [7]. Lam and Corya et al. reported that antidepressants that enhancing DA neurotransmission may attenuate the persistent anhedonic state found in many depressed patients [8], [9]. Multiple studies have demonstrated that the adjunctive use of psychostimulants accelerate the response to SSRI/SNRI treatment [8], [10], [11]. Several studies have suggested that enhancing DA neurotransmission reduces SSRIs/SNRIs-induced sexual dysfunction and sleepiness [12], [13]. Moreover, anatomical and functional evidence of interactions between monoaminergic neurons also exists [14]–[16]. Hence, putative antidepressants, which inhibit DA, 5-HT and NE transporters named as triple reuptake inhibitors (TRIs), are an attractive pharmacotherapeutic approach for depression compared with currently available antidepressants.

In this paper, we examine 4-(2-(dimethylamino)-1-(1-hydroxycyclohexyl) ethyl) phenyl 4-methylbenzoate hydrochloride (LPM570065), a novel TRI that is also designed as a methyl benzoate of desvenlafaxine and converts to desvenlafaxine under the hydrolysis of ubiquitous esterases *in vivo*. Desvenlafaxine is an SNRI that increases extracellular 5-HT and NE levels by inhibiting the reuptake of 5-HT and NE. Although desvenlafaxine is used clinically, it has an insufficient antidepressant effect, a high risk of side effects and relatively low barrier permeability [17], [18]. The IC_50_ values of desvenlafaxine inhibition of 5-HT and NE reuptake *in vitro* are 53 and 538 nM, respectively [17]. In comparison, the IC_50_ values of LPM570065 inhibition of 5-HT, DA and NE reuptake *in vitro* are 723, 491 and 763 nM, respectively. TRIs such as LPM570065 may have better barrier permeability, and with the addition of inhibiting DA reuptake, better efficacy in increasing monoamine neurotransmission may occur.

The striatum, which contains very dense dopaminergic neurons, is a major area for the convergence of dopaminergic and serotonergic inputs from the midbrain [19]. The output of the striatum is directed to the globus pallidus internus [20], [21], and the globus pallidus internus projects densely to the lateral habenula, which may be involved in the response to stress, nociception, circadian rhythms, learning, reward and the development of major depression [22]–[28]. Based on the monoaminergic networks, the present study was designed to investigate the acute and chronic effects of LPM570065 and desvenlafaxine on the extracellular levels of 5-HT, DA and NE in the rat striatum and to characterise their mechanisms of action in combination with WAY-100635 (a 5-HT_1A_ receptor antagonist). Forced swim test was also introduced to evaluate the antidepressant-like activity of LPM570065 compared with desvenlafaxine. In addition, the concentrations of LPM570065 and desvenlafaxine in the rat striatum were examined. Different pharmacokinetic profiles of LPM570065 in the rat striatum may produce different effects on monoamine neurotransmission due to its complex metabolic process, and variable administration routes of LPM570065 were used in the present study. This work may help us to better understand the mechanisms of action of LPM570065 and develop the optimal pharmaceutical formulation of LPM570065.

## Materials and Methods

### Ethics Statement

Adult male Sprague-Dawley rats (weighing 250–300 g; Vital River, Beijing, China) were used in the present study. All animals were group-housed in a room at 22±2°C and a relative humidity of 50±5% under a reversed 12 h light-dark cycle (lights on at 7∶00 am). Tap water and food (Normal chow; Trophic Co., Nantong, China) were available *ad libitum*. All experiments were performed upon approval from the Institutional Animal Care and Use Committee of Jilin University (Permit Number: 2010–069) and State Key Laboratory of Long-acting and Targeting Drug Delivery System (Permit Number: 2009-0009) and complied with the guidelines of the National Institute of Health Guide for the Care and Use of Laboratory Animals (NIH publication no. 80-23). All efforts were made to minimise animal suffering and the number of animals used for all studies.

### Chemicals

**Figure 1:**
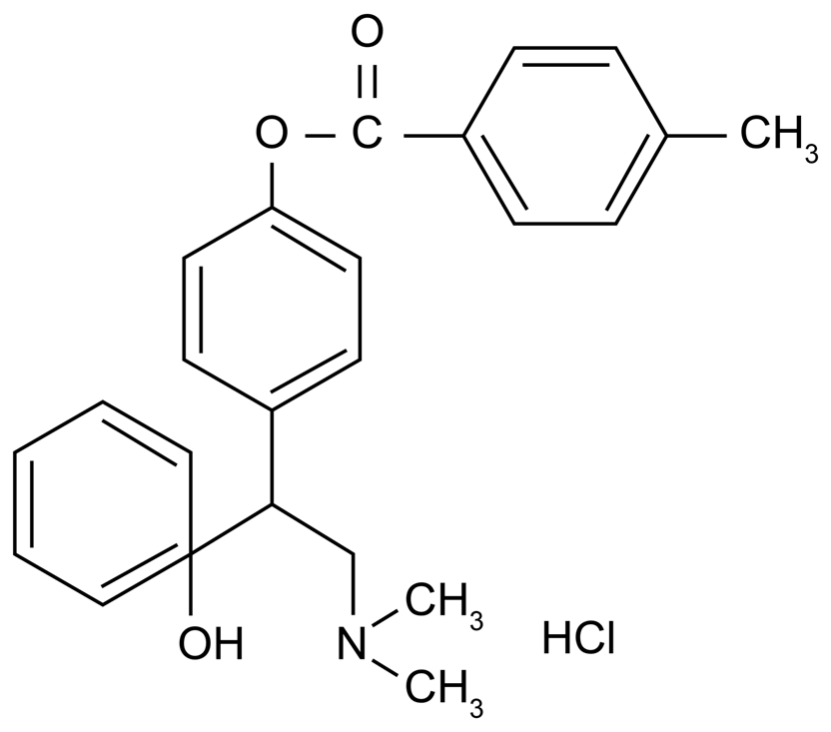
The chemical structure of LPM570065.

LPM570065 (Figure 1), desvenlafaxine and 0.5% carboxymethylcellulose sodium were provided by the State Key Laboratory of Long-acting and Targeting Drug Delivery Technologies (Yantai, China). Deionised water was provided from a MilliQ water purification system (Millipore Co.; Massachusetts, USA). DA, 5-HT, NE, WAY-100635, raclopride and the chemicals used to prepare artificial cerebrospinal fluid, including sodium chloride, potassium chloride and calcium chloride, were purchased from Sigma-Aldrich (St. Louis, MO, USA). Potassium ferricyanide, sodium dihydrogen phosphate and CAPS were obtained from Aladdin (Shanghai, China). Glycine and diphenylethylenediamine were purchased from TCI (Tokyo, Japan). Benzylamine hydrochloride was obtained from J&K CHEMICA (Beijing, China). Chloral hydrate was purchased from Tianjin Fuchen Chemical Reagents Factory (Tianjin, China). Sodium acetate trihydrate was obtained from Kermel (Tianjin, China). Disodium EDTA was purchased from Tianjin Beichen District Reagents Factory (Tianjin, China). Methanol and acetonitrile were obtained from MERCK (Darmstadt, Germany). Glucose injections were purchased from Shandong Kelun Pharmaceutical Company (Binzhou, China). Benzylpenicillin sodium for injection was obtained from Shandong Lukang Pharmaceutical Company (Jining, China).

### Manipulations of the Test Substances

The dosages of LPM570065 and desvenlafaxine used with different administration routes referred to previous studies described by Tian et al. and Alfinito et al. [17], [29], [30] and was determined by preliminary experiments. Oral solutions of LPM570065 and desvenlafaxine for intragastric administration (0.06 mmol·kg^−1^ p.o.) were prepared by dissolving the compounds in 10% glucose. Oral suspensions of LPM570065 and desvenlafaxine for intragastric administration (0.06 mmol·kg^−1^ p.o.) were prepared by suspending the compounds in 0.5% carboxymethylcellulose sodium. Intravenous solutions of LPM570065 and desvenlafaxine for intravenous administration (0.04 mmol·kg^−1^ i.v.) were prepared by dissolving the compounds in 10% glucose. WAY-100635 was dissolved in 0.9% saline and was administered subcutaneously (0.3 mg·kg^−1^ s.c.). Raclopride was dissolved in 0.9% saline and was administered subcutaneously (0.5 mg·kg^−1^ s.c.). Different vehicles were used for administration using different administration routes.

### High Performance Liquid Chromatography (HPLC) Determination of LPM570065 and Desvenlafaxine Concentrations in the Rat Striatum

Animals were randomly divided into six groups. Animals in Group 1, 2 and 3 were administered oral solution, oral suspension and intravenous solution of LPM570065, respectively. Animals in Group 4, 5 and 6 were administered oral solution, oral suspension and intravenous solution of desvenlafaxine, respectively. Food was restricted from 12 h pre-dosing to 60 min post-dosing. At 0.05, 0.25, 0.5, 1, 2 and 4 h post-dosing, the animals (n = 6 rats per time point in each group) were anesthetised with 10% chloral hydrate, and then were perfused with 40 mL of phosphate-buffered saline (4°C) through the left ventricle. The striatum was dissected, placed in 0.25 mL of ice-cold acetonitrile/deionised water (2∶3) and maintained on ice until homogenisation. Striata were homogenised for 45 s and sonicated for 10 s. The experimental sample and calibration sample were obtained by adding 200 µL of sample or control matrix, respectively, into the tube. Calibration samples for standard curves (50–40,000 ng·mL^−1^ for LPM570065; 10–4,000 ng·mL^−1^ for desvenlafaxine) were further prepared by adding 10 µL of the appropriate concentration of LPM570065 and desvenlafaxine. Protein was then precipitated by the addition of 800 µL of acetonitrile and centrifugation at 13,000 rpm for 10 min. The supernatant was transferred and evaporated to dry particles under nitrogen atmosphere at 40°C. The resulting material was reconstituted in 0.1 M NH_4_PO_3_: acetonitrile solution (3∶1, 200 µL) and then centrifuged at 13,000 rpm for 10 min. 40 µL of the solution was then subjected to HPLC (Agilent Co., Palo Alto, USA) using an Ultimate XB-C18 column (4.6×250 mm, 5 µm, Welch Materials Co., Maryland, USA) maintained at 30°C and a VWD set at 230 nm. The mobile phase (0.1% TFA, 30% acetonitrile) was delivered at a flow rate of 1.0 mL·min^−1^. The detection limit (at a signal-to-noise ratio of 3) for LPM570065 and desvenlafaxine were 10.0 ng·mL^−1^ and 2.0 ng·mL^−1^, respectively.

### Microdialysis Study: Determination of the Extracellular 5-HT, DA and NE Levels in the Rat Striatum After Acute and Chronic Administration of LPM570065 and Desvenlafaxine

#### Procedures

**Figure 2:**
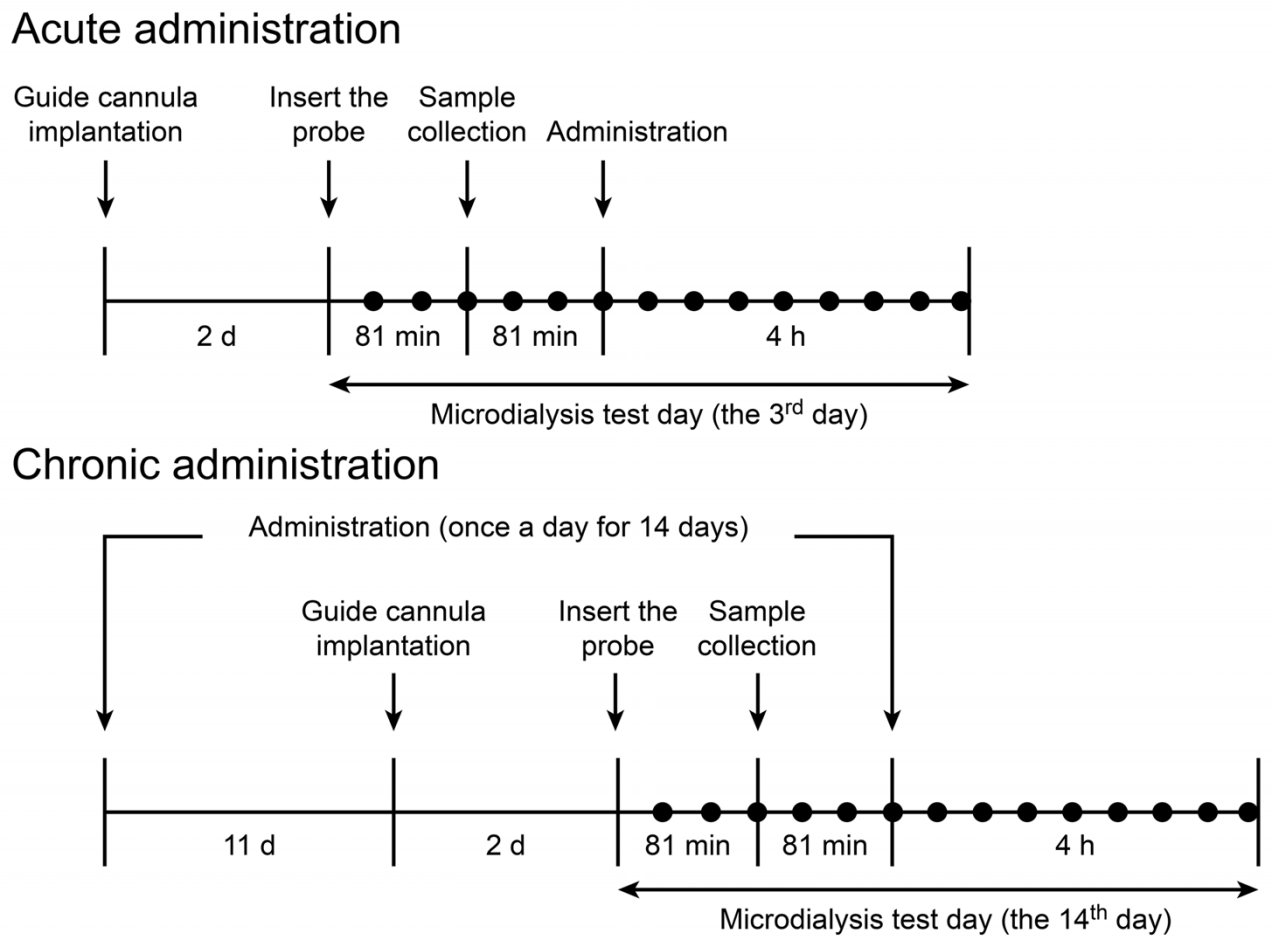
The microdialysis study design after acute and chronic drug administration.

The microdialysis study design is shown in Figure 2. For acute administration, drug-naive animals were randomly divided into nine groups (n = 8 rats for each group). Acute administration to examine the effects of LPM570065 and desvenlafaxine on extracellular 5-HT, DA and NE levels was performed by administering oral solutions, oral suspensions and intravenous solutions of LPM570065 and desvenlafaxine (Table 1). An equal number of animals were used to examine the acute effects of LPM570065 and desvenlafaxine on extracellular 5-HT levels under the blockade of 5-HT_1A_ receptors by pretreatment with WAY-100635. Animal grouping and administration were the same as above expect WAY-100635 was administered 20 min prior to LPM570065 and desvenlafaxine. The acute effects of intravenous administration of LPM570065 on extracellular 5-HT levels under the blockade of D_2_ receptors were also examined by pretreatment with raclopride (20 min prior to LPM570065). For the 14-day chronic administration, animals were randomly divided into three groups (n = 8 rats for each group). Oral suspensions of LPM570065, desvenlafaxine and vehicle were administered daily for 14 days (Table 1). On the 14^th^ day of chronic administration, the effects of LPM570065 and desvenlafaxine on the extracellular 5-HT, DA and NE levels were examined.

**Table 1:** Basal extracellular levels of 5-HT, DA and NE.

				Basal monoamine levels (mean±SD)		
Treatment	Administration route and dosage	Substance	Group	5-HT (nM)	DA (nM)	NE (nM)
Acute	Oral solution (0.06 mmol·kg^−1^ p.o.)	LPM570065	1^a^	4.0±1.6	6.9±1.4	1.1±0.5
		Desvenlafaxine	2^a^	4.1±1.6	6.6±1.5	1.2±0.3
		Vehicle	3^a^	3.6±2.5	7.9±1.8	1.2±0.4
	Oral suspension (0.06 mmol·kg^−1^ p.o.)	LPM570065	4^a^	4.3±0.9	7.7±1.7	1.1±0.4
		Desvenlafaxine	5^a^	4.3±1.4	7.2±1.5	1.0±0.4
		Vehicle	6^a^	3.6±1.7	8.1±2.5	1.1±0.5
	Intravenous solution (0.04 mmol·kg^−1^ i.v.)	LPM570065	7^b^	3.7±1.6	7.7±1.0	1.3±0.4
		Desvenlafaxine	8^a^	4.4±1.9	7.6±1.4	1.2±0.4
		Vehicle	9^a^	3.4±1.4	6.9±1.8	1.2±0.4
Chronic	Oral suspension (0.06 mmol·kg^−1^·day^−1^ p.o.)	LPM570065	1^a^	3.4±1.3	6.4±1.4	1.1±0.4
		Desvenlafaxine	2^a^	4.1±1.9	6.7±1.4	1.2±0.5
		Vehicle	3^b^	3.8±1.7	7.1±1.3	1.1±0.3

a n = 8 adult male Sprague-Dawley rats.

b n = 7 adult male Sprague-Dawley rats.

#### Guide cannula implantation

Animals were anaesthetised with 10% chloral hydrate and secured in a stereotaxic frame (David Kopf Instruments, CA, USA), and their body temperature was maintained at 37±1°C. After exposure of the skull, a hole for the probe was drilled. The CMA/12 guide cannula (CMA/Microdialysis, Stockholm, Sweden) was implanted into the left striatum (A = 0.2 mm, L = 3.0 mm and V = −3.2 mm from the bregma and the dural surface) [31] and fixed firmly to the skull surface. Immediately after surgery, the animals were individually housed in Plexiglas cages (45 cm^2^) with free access to food and water and were allowed 2 d of postoperative recovery before the microdialysis test.

#### Microdialysis test

The CMA/12 microdialysis probe (4 mm, length, cut-off 20 kDa) was perfused with artificial cerebrospinal fluid (145 mM NaCl, 2.7 mM KCl and 1.2 mM CaCl_2_, pH 7.4) at a flow rate of 1.5 µL·min^−1^ for at least 2 h before the experiment, and then the probe was inserted into the guide cannula implanted in rat striatum. The samples were collected in 27-min intervals. The first three samples were discarded and the subsequent three samples were collected to analyse the basal extracellular levels of 5-HT, DA and NE. After drug administration, the samples were collected for an additional 160 or 240 min.

#### HPLC measurement of 5-HT, DA and NE

The concentrations of 5-HT, DA and NE in microdialysis samples were determined by HPLC with fluorescence detection following derivation as previously described [32], [33]. Briefly, the derivation reagent solution for determining 5-HT and NE concentrations was a mixture containing 0.5 M benzylamine, 0.3 M CAPS buffer (pH 12.0), 10 mM potassium ferricyanide and methanol (1∶1:1∶12, v v^−1^ v^−1^ v^−1^). The derivation reagent solution for determining DA concentrations was a mixture containing 0.1 M diphenylethylenediamine and 0.3 M glycine (2∶1, v v^−1^). 20 µL of the derivation reagent solution was added to a vial containing 20 µL of the microdialysis sample or standard solution. The vial was heated at 50°C for 20 min. After being cooled in water, 20 µL of the final reaction mixture was subjected to HPLC using the Ultimate XB-C18 column maintained at 23–25°C and a FLD set at 345 nm for the excitation wavelength and 480 nm for the emission wavelength. The mobile phase included a mixture of acetonitrile and acetate buffer (15 mM, pH 5.0, containing 1 mM disodium EDTA) at a ratio of 31∶69 (v v^−1^). The detection limit (at a signal-to-noise ratio of 3) was 0.25 nM for NE and 5-HT and 1.0 nM for DA.

### Forced Swim Test

Forced swim test was used to measure the depression-like behavior in rats after acute and chronic drug treatment. Animals were randomly divided into six groups. The animals in Groups 1, 2 and 3 were used for acute treatment, and the animals in Groups 4, 5 and 6 were used for chronic treatment (once a day for 14 days). The acute and chronic treatments were performed by providing animals with oral suspensions of LPM570065, desvenlafaxine and vehicle. We followed a previously established protocol to conduct the forced swim test [34]. The entire test was performed in 2 consecutive days: a 15-min pretest session on day 1 followed by a 5-min test session the next day. On the pretest day, rats were individually forced to swim for 15 min in a plastic cylinder (diameter 22 cm, height 46 cm) containing water (25±1°C) with a depth of 30 cm. On the test day, rats were placed in the cylinder again for a 5-min test trial at 0.5, 1, 2 and 4 h post-dosing (n = 10 rats per time point in each group). The total duration of immobilization was recorded. The criterion for immobilization was vertical floating in the water and movements necessary to keep the head above the water. After the test, animals were removed and dried with towels before returning to their home cage.

### Data Analysis

All values are expressed as the mean±SD (n = 6–10 rats per time point). For analysing the data of LPM570065 and desvenlafaxine concentrations in the rat striatum, the total exposure of LPM570065 and desvenlafaxine in rat striatum over a 4-h post-dosing period was expressed as the area under the curve (AUC_0–240 min_), and the values of AUC_0–240 min_ were calculated. For the microdialysis study, the extracellular monoamine levels (5-HT, DA and NE) in three baseline dialysis samples were averaged and the post-drug monoamine levels were calculated as a percentage change from the mean basal levels (taken as 100%) for each animal [35]. SPSS 13.0 software (SPSS Inc., IL, USA) was used for the following data analysis. The extracellular levels of monoamines after administration were analysed with respect to the treatment group (between-subject factor) and the interaction between group and time using two-way analysis of variance (ANOVA) with repeated measures. Significant effects of treatment group at a given time-point were further analysed by using Bonferroni’s post-test where appropriate. The overall effects of LPM570065 and desvenlafaxine on the extracellular monoamine levels are presented as the area under the curve (AUC_0-t_) calculated as the sum of relative changes in the extracellular monoamine levels over the t min post-treatment period and subtracted from the mean AUC_0-t_ value of the vehicle-treated group [36]. Separate one-way ANOVAs were used to measure the overall effect of drug treatment. For the forced swim test, the data were analyzed by one-way ANOVA with Bonferroni’s post-test. A level of *p*<0.05 was accepted as a significant effect.

## Results

### The LPM570065 and Desvenlafaxine Concentration in the Rat Striatum After Using Different Administration Routes

**Figure 3:**
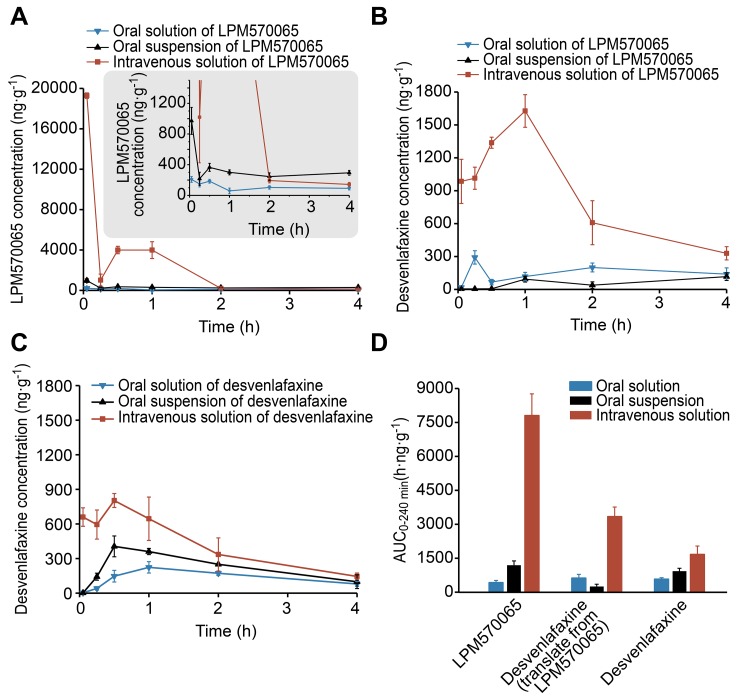
LPM570065 and desvenlafaxine concentrations in the rat striatum after administration. A single dose of LPM570065 or desvenlafaxine was administered by using different administration routes (blue: 0.06 mmol·kg^−1^ for oral solution; black: 0.06 mmol·kg^−1^ for oral suspension; red: 0.04 mmol·kg^−1^ for intravenous solution). All data are presented as the mean±SD, n = 6 rat per time point. (A) LPM570065 concentrations after administration of LPM570065 using different administration routes. The small graphic in Figure 3A presents a partially enlarged detail of the LPM570065 concentrations using different administration routes. (B) Desvenlafaxine concentrations after LPM570065 administration using different administration routes. (C) Desvenlafaxine concentrations after the administration of desvenlafaxine using different administration routes. (D) Total exposure of LPM570065 and desvenlafaxine in the rat striatum over a 4-h period after administration of LPM570065 or desvenlafaxine using different administration routes. Total exposure is expressed as the area under the curve (AUC_0–240 min_) of LPM570065 and desvenlafaxine in the form of a histogram.

The LPM570065 and desvenlafaxine concentrations in the rat striatum were determined for each time point over a 4-h period, as shown in Figure 3. LPM570065 was converted to desvenlafaxine *in vivo* after administration. LPM570065 and desvenlafaxine coexisted for a period of time and presented a dynamic change in the rat striatum (Figure 3A and 3B). The cumulative LPM570065 and desvenlafaxine AUC_0–240 min_ values after treatment with LPM570065 were greatest using intravenous solution, followed by oral suspension and oral solution as the administration routes (Figure 3D). The results clearly showed that LPM570065 given using different administration routes exhibited larger total exposure (expressed as AUC_0–240 min_ value) compared with the relative administration of desvenlafaxine (Figure 3D).

### Basal Extracellular Levels of 5-HT, DA and NE

The average baseline concentrations of extracellular 5-HT, DA and NE in the rat striatum (not corrected for *in vitro* dialysis probe recovery) are presented in Table 1. No significant differences were observed in the basal extracellular 5-HT, DA and NE levels between the treatment groups. The basal extracellular 5-HT levels, but not the NE and DA levels, decreased gradually during the experimental period, so animals were tested in a quiet environment to reduce the volatility of extracellular 5-HT levels.

### The Effects of LPM570065 and Desvenlafaxine on the Extracellular 5-HT Levels After Acute Administration Using Different Administration Routes in Combination with WAY-100635

**Figure 4:**
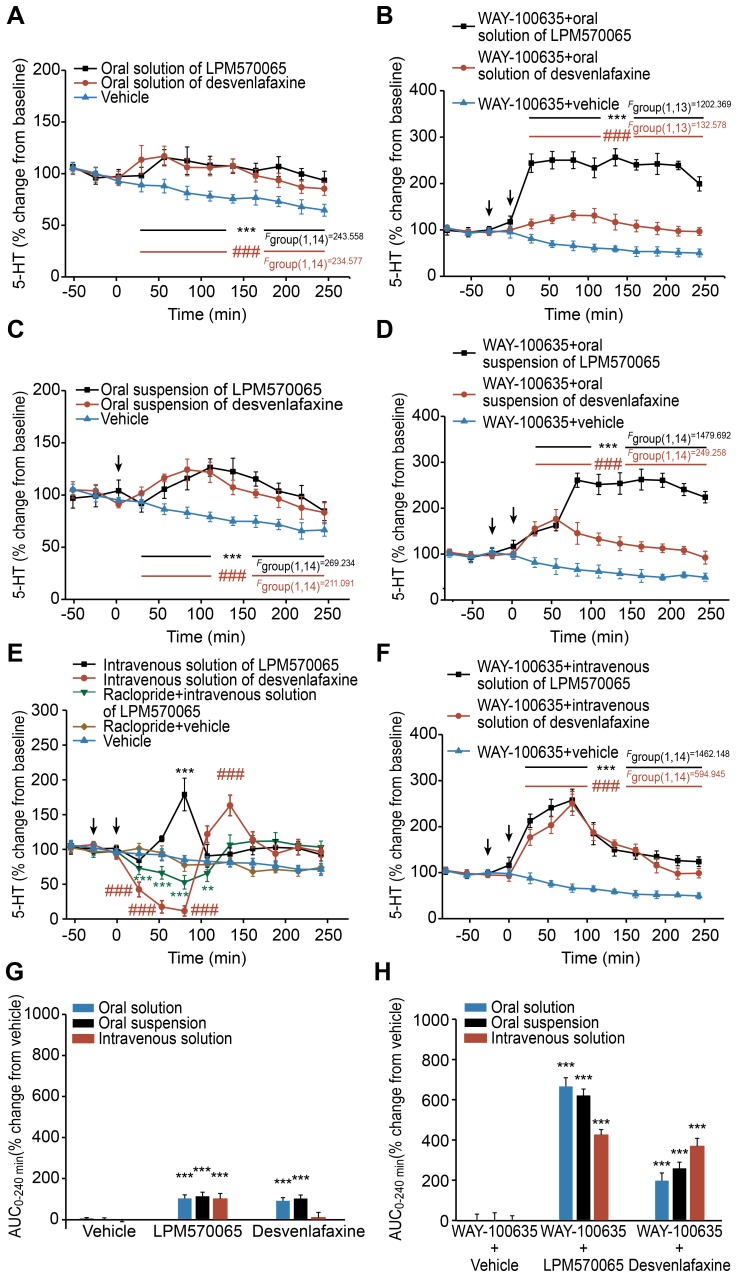
The effects of LPM570065, desvenlafaxine and vehicle on extracellular 5-HT levels after acute administration. The effects of LPM570065 (black line), desvenlafaxine (red line) and vehicle (blue line) alone on extracellular 5-HT levels in the rat striatum after acute administration using different administration routes are indicated as follows: (A) for oral solution, (C) for oral suspension and (E) for intravenous solution; the effects of WAY-100635 plus LPM570065 (black line), desvenlafaxine (red line) or vehicle (blue line) on the extracellular 5-HT levels in the rat striatum after acute administration using different administration routes are indicated as follows: (B) for oral solution, (D) for oral suspension and (F) for intravenous solution. The effects of raclopride plus intravenous administration of LPM570065 on extracellular 5-HT levels in the rat striatum are also presented in Figure 4E. WAY-100635 (0.3 mg·kg^−1^ s.c.) or raclopride (0.5 mg·kg^−1^ s.c.) was administered at time −20 min; LPM570065, desvenlafaxine (0.06 mmol·kg^−1^ for oral solution; 0.06 mmol·kg^−1^ for oral suspension; 0.04 mmol·kg^−1^ for intravenous solution) or the appropriate vehicle was administered at time 0 min. Data are presented as the mean±SD, n = 7–8; ***p<0.001, **p<0.01 LPM570065 vs. relative vehicle; *^###^p*<0.001 desvenlafaxine vs. relative vehicle; two-way ANOVA with repeated measures, Bonferroni’s post-test. The overall effects on extracellular 5-HT levels in the rat striatum following treatment with LPM570065, desvenlafaxine or vehicle alone and WAY-100635 plus LPM570065, desvenlafaxine or vehicle are indicated in (G) and (H), respectively (blue column: oral solution; black column: oral suspension; red column: intravenous solution). The columns represent the AUC_0–240 min_ values calculated as the differences in relative changes in extracellular 5-HT levels over a 4-h period between the drug-treated and relative vehicle-treated groups. Data are presented as the mean±SD, n = 7–8; ***p<0.001 drug vs. relative vehicle; one-way ANOVA.

The extracellular 5-HT levels in the rat striatum were determined for each time point over a 4-h post-dosing period. Figure 4A and 4C show that the oral administration of LPM570065 (*p*<0.001) or desvenlafaxine (*p*<0.001) caused slight but significant increases in the extracellular 5-HT levels compared with the relative vehicle group. As shown in Figure 4E, a sharp peak (177±23% above basal levels) was observed at 90 min after the intravenous administration of LPM570065. However, the intravenous administration of desvenlafaxine induced a marked 90% decrease in the extracellular 5-HT levels during the first 90 min of the post-dosing period; the 5-HT levels increased to 162±15% and then returned to basal levels soon afterwards. Figure 4E also shows that the increase in extracellular 5-HT levels within the first 120 min after intravenous administration of LPM570065 was reduced in the presence of raclopride and that the reduction in 5-HT levels was less than the extent by intravenous administration of desvenlafaxine. The overall effects of LPM570065 and desvenlafaxine on the 5-HT AUC_0–240 min_ values are presented in Figure 4G. The oral and intravenous administration of LPM570065 caused significant increases in the 5-HT AUC_0–240 min_ values (*p*<0.001); however, only the oral administration of desvenlafaxine induced significant increases in the 5-HT AUC_0–240 min_ values (*p*<0.001).

As shown in Figure 4B, the LPM570065 oral solution in combination with WAY-100635 (a 5-HT_1A_ receptor antagonist) caused a marked increase in the extracellular 5-HT levels, which was significantly higher (*F*_group(1,14)_ = 536.776, *p*<0.001) than the levels induced in the relative WAY-100635+desvenlafaxine group. The LPM570065 oral suspension in combination with WAY-100635 induced a slight increase in extracellular 5-HT levels in the first hour and then a marked increase, which was still significantly higher (*F*_group(1,14)_ = 544.427, *p*<0.001, *F*_group×time(8,112)_ = 78.051, *p*<0.001) than the levels induced in the relative WAY-100635+desvenlafaxine group (Figure 4D). Figure 4F shows that the intravenous solutions of LPM570065 and desvenlafaxine in combination with WAY-100635 induced similar increases in extracellular 5-HT levels, which were significantly higher (*p*<0.001) than the levels induced in the relative WAY-100635+vehicle group. The peak values of 5-HT induced by LPM570065 were similar between different administration routes. These results demonstrated that the acute administration of LPM570065 in combination with WAY-100635 exhibited a ceiling effect on increasing the extracellular 5-HT levels compared with the fully dose-dependent effect of WAY-100635+desvenlafaxine on the 5-HT levels. The overall effects of LPM570065 and desvenlafaxine in combination with WAY-100635 on the 5-HT AUC_0–240 min_ values are presented in Figure 4H. For the WAY-100635+LPM570065 groups, the 5-HT AUC_0–240 min_ value after intravenous administration, compared with the values after oral administration, were significantly reduced (*p*<0.001) from 652±42% (oral solution) and 608±31% (oral suspension) to 415±26% (intravenous solution). For the WAY-100635+desvenlafaxine groups, the 5-HT AUC_0–240 min_ values changed proportionally in response to the total exposure of desvenlafaxine (Figure 3D and 4H). Figure 4G and 4H show that the overall effects on the extracellular 5-HT levels after treatment with WAY-100635+LPM570065/desvenlafaxine were significantly (*p*<0.001) higher compared with the effects induced by treatment with LPM570065/desvenlafaxine alone.

### The Effects of LPM570065 and Desvenlafaxine on the Extracellular DA Levels After Acute Administration Using Different Administration Routes

**Figure 5:**
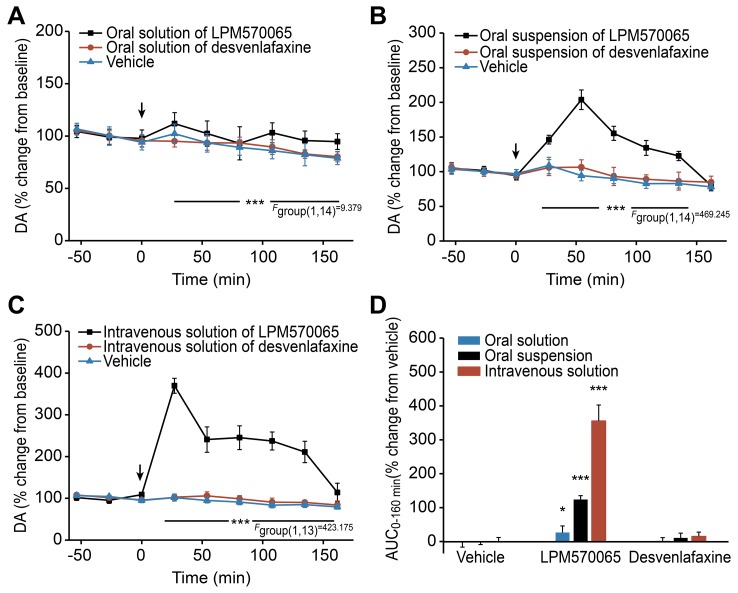
The effects of LPM570065, desvenlafaxine and vehicle on the extracellular DA levels after acute administration. The effects of LPM570065 (black line), desvenlafaxine (red line) and vehicle (blue line) on the extracellular DA levels in the rat striatum after acute administration using different administration routes are indicated as follows: (A) for oral solution, (B) for oral suspension and (C) for intravenous solution. LPM570065 and desvenlafaxine (0.06 mmol·kg^−1^ for oral solution; 0.06 mmol·kg^−1^ for oral suspension; 0.04 mmol·kg^−1^ for intravenous solution) or the appropriate vehicle was administered at time 0 min. Data are presented as the mean±SD, n = 7–8; ***p<0.001 LPM570065 vs. relative vehicle; two-way ANOVA with repeated measures. (D) The overall effects on the extracellular DA levels in the rat striatum following treatment with LPM570065, desvenlafaxine or vehicle using different administration routes (blue column: oral solution; black column: oral suspension; red column: intravenous solution). The columns represent the AUC_0–160 min_ values calculated as the differences in relative changes in the extracellular DA levels over a 2.7-h period between the drug-treated and relative vehicle-treated groups. Data are presented as the mean±SD, n = 7–8; ***p<0.001, *p<0.05 LPM570065 vs. relative vehicle; one-way ANOVA.

The extracellular DA levels in the rat striatum were determined for each time point over a 2.7-h post-dosing period. As shown in Figure 5A, 5B and 5C, desvenlafaxine did not significantly alter the extracellular DA levels, either by oral administration or intravenous administration. Figure 5A shows that the LPM570065 oral solution induced a slight but significant increase in the extracellular concentrations of DA (*p*<0.001 compared with the vehicle group). The LPM570065 oral suspension caused a significant increase in the extracellular DA levels (*p*<0.001 compared with the vehicle group), with a peak value of 194±13% at 60 min (Figure 5B). The intravenous administration of LPM570065 induced a massive and significant increase in the extracellular DA levels (*p*<0.001 compared with the vehicle group), reaching the peak level of 343±16% at 30 min (Figure 5C). The overall effects of LPM570065 and desvenlafaxine on the relative DA AUC_0–240 min_ values are presented in Figure 5D. The oral solution, oral suspension and intravenous solution of LPM570065 increased the DA AUC_0–160 min_ values to 26±19%, 123±11% (*p*<0.001) and 356±47% (*p*<0.001) of the relative vehicle groups, respectively.

### The Effects of LPM570065 and Desvenlafaxine on the Extracellular NE Levels After Acute Administration Using Different Administration Routes

**Figure 6:**
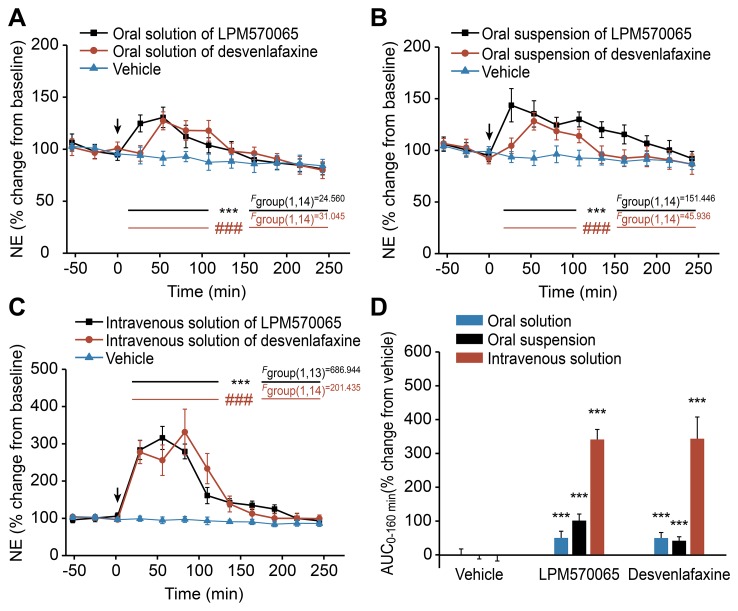
The effects of LPM570065, desvenlafaxine and vehicle on the extracellular NE levels after acute administration. The effects of LPM570065 (black line), desvenlafaxine (red line) and vehicle (blue line) on the extracellular NE levels in the rat striatum after acute administration using different administration routes are indicated as follows: (A) for oral solution, (B) for oral suspension and (C) for intravenous solution. LPM570065, desvenlafaxine (0.06 mmol·kg^−1^ for oral solution; 0.06 mmol·kg^−1^ for oral suspension; 0.04 mmol·kg^−1^ for intravenous solution) or the appropriate vehicle was administered at time 0 min. Data are presented as the mean±SD, n = 7–8; ***p<0.001 LPM570065 vs. relative vehicle; *^###^p*<0.001 desvenlafaxine vs. relative vehicle; two-way ANOVA with repeated measures. (D) The overall effects on the extracellular NE levels in the rat striatum following treatment with LPM570065, desvenlafaxine or vehicle using different administration routes (blue column: oral solution; black column: oral suspension; red column: intravenous solution). The columns represent the AUC_0–240 min_ values calculated as the differences in relative changes in extracellular NE levels over a 4-h period between the drug-treated and relative vehicle-treated groups. Data are presented as the mean±SD, n = 7–8; ***p<0.001 drug vs. relative vehicle; one-way ANOVA.

The extracellular NE levels in the rat striatum were determined for each time point over a 4-h post-dosing period. As shown in Figure 6A and 6B, the oral administration of LPM570065 (*p*<0.001) and desvenlafaxine (*p*<0.001) induced slight but significant increases in the extracellular NE levels compared with the relative vehicle group. Figure 6C shows that intravenous administration of LPM570065 (*p*<0.001) and desvenlafaxine (*p*<0.001) induced marked and significant increases in the extracellular NE levels compared with relative vehicle group. The overall effects of LPM570065 and desvenlafaxine on the relative NE AUC_0–240 min_ values are presented in Figure 6D. Administration of LPM570065 via an oral solution, oral suspension or intravenous solution increased the NE AUC_0–160 min_ values to 49±20% (*p*<0.001), 100±19% (*p*<0.001) and 340±30% (*p*<0.001) of the relative vehicle groups, respectively. The NE AUC_0–240 min_ values were not significantly different between the LPM570065-treated groups and the relative desvenlafaxine-treated groups, except for the oral suspensions of LPM570065 and desvenlafaxine (*p*<0.001).

### The Effects of LPM570065 and Desvenlafaxine on the Extracellular 5-HT, DA and NE Levels After Chronic Administration

**Figure 7:**
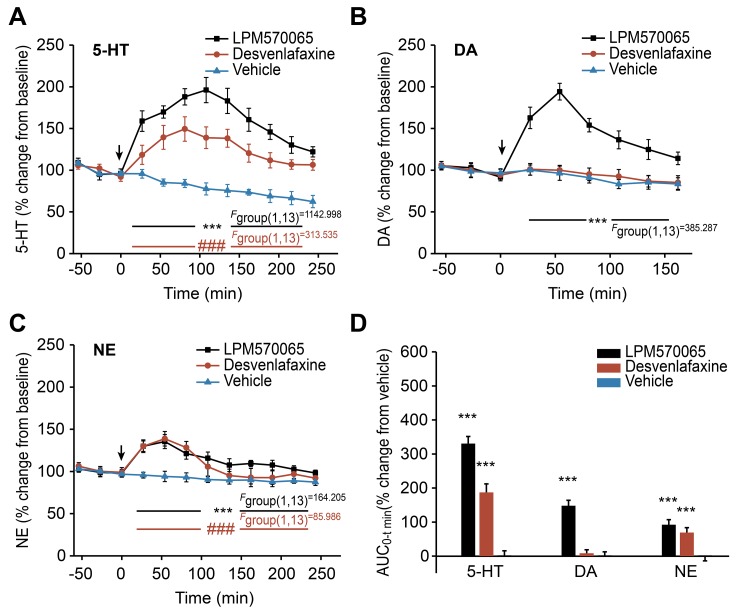
The effects of LPM570065, desvenlafaxine and vehicle on the extracellular monoamine levels after chronic administration. The effects of LPM570065 (black line), desvenlafaxine (red line) and vehicle (blue line) on the extracellular 5-HT, DA and NE levels in the rat striatum after chronic administration (oral suspension: 0.06 mmol·kg^−1^·day^−1^ for 14 days) are indicated in (A), (B) and (C), respectively. LPM570065, desvenlafaxine or vehicle was administered at time 0 min. Data are presented as the mean±SD, n = 7–8; ***p<0.001 LPM570065 vs. vehicle; *^###^p*<0.001 desvenlafaxine vs. vehicle; two-way ANOVA with repeated measures. (D) The overall effects on the extracellular 5-HT, DA and NE levels in the rat striatum following the chronic treatment with LPM570065 (black column), desvenlafaxine (red column) or vehicle (blue column). The columns represent the AUC_0-t min_ values calculated as the differences in relative changes in the extracellular monoamine levels over a period between the drug-treated and vehicle-treated groups (t = 240 min for 5-HT and NE; t = 160 min for DA). Data are presented as the mean±SD, n = 7–8; ***p<0.001 drug vs. vehicle; one-way ANOVA.

After a 14-day chronic treatment period with LPM570065/desvenlafaxine, the extracellular 5-HT, DA and NE levels in the rat striatum were determined for each time point. As shown in Figure 7A, both LPM570065 (*p*<0.001) and desvenlafaxine (*p*<0.001) had significant enhancing effects on the extracellular concentrations of 5-HT compared with the vehicle. The overall effects of LPM570065 and desvenlafaxine on the relative 5-HT AUC_0–240 min_ values are presented in Figure 7D. LPM570065 and desvenlafaxine caused significant increases in the 5-HT AUC_0–240 min_ values by 331±21% (*p*<0.001) and 187±25% (*p*<0.001) of the vehicle group, respectively. The overall effects on the extracellular 5-HT levels after treatment with LPM570065 were significantly higher (*p*<0.001) compared with the effects induced by treatment with desvenlafaxine.

As shown in Figure 7B, the chronic administration of LPM570065 caused a significant increase in extracellular DA levels (*p*<0.001 compared with the vehicle group), reaching a peak value of 194±10% at 60 min; however, the chronic administration of desvenlafaxine did not significantly alter the extracellular DA levels. The overall effects of LPM570065 and desvenlafaxine on the relative DA AUC_0–240 min_ values are presented in Figure 7D. LPM570065 increased the DA AUC_0–160 min_ value to 148±16% (*p*<0.001) of the vehicle group.

The chronic administration of LPM570065 caused a significant increase in the extracellular NE levels (*p*<0.001 compared with the vehicle group), reaching a peak value of 135±8% at 60 min, and desvenlafaxine induced a significant increase (*p*<0.001 compared with the vehicle group) to 139±9% at 60 min (Figure 7C). Compared with desvenlafaxine, LPM570065 demonstrated a prolonged effect on the NE levels. As shown in Figure 7D, both LPM570065 (*p*<0.001) and desvenlafaxine (*p*<0.001) significantly increased the NE AUC_0–160 min_ values compared with the vehicle group. The overall effects of LPM570065 on the extracellular NE levels were significantly greater (*p*<0.01) than the overall effects of desvenlafaxine.

### The Effects of LPM570065 and Desvenlafaxine on the Forced Swim Test after Acute and Chronic Administration

**Figure 8:**
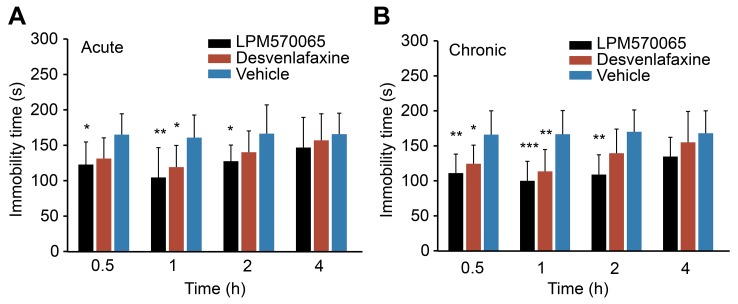
Forced swim test in rats for evaluating antidepressant-like activity of LPM570065 and desvenlafaxine. The effects of LPM570065 (black column), desvenlafaxine (red column) and vehicle (blue column) on the immobility time in the forced swim test over a 4-h post-dosing period are indicated as follows: (A) for acute administration and (B) for chronic administration. Acute (0.06 mmol·kg^−1^) and chronic (0.06 mmol·kg^−1^·day^−1^ for 14 days) administration were performed by providing rats the oral suspensions of LPM570065 and desvenlafaxine. At 0.5, 1, 2 and 4 h post-dosing, rats were placed in the cylinder again for 5 min (test session), and the total duration of immobilization was recorded. Data are presented as the mean±SD (n = 10) in the form of a histogram; ***p<0.001, **p<0.01, *p<0.05 drug vs. relative vehicle; one-way ANOVA, Bonferroni’s post-test.

The immobility time data in the forced swim test after acute and chronic drug treatments are shown in Figure 8A and 8B, respectively. Both acute and chronic administration of LPM570065 significantly reduced the immobility time at 0.5, 1 and 2 h post-dosing compared with the relative vehicle groups (for the acute administration: *p*<0.05, *p*<0.01 and *p*<0.05, respectively; for the chronic administration: *p*<0.01, *p*<0.001 and *p*<0.01, respectively). Desvenlafaxine only significantly reduced the immobility time at 1 h (*p*<0.05) after the acute administration and at 0.5 (*p*<0.05) and 1 h (*p*<0.01) after the chronic administration. Both acute and chronic administration of LPM570065 induced more reduction of the immobility time compared with the relative administration of desvenlafaxine. These results demonstrated that LPM570065 exhibited a better antidepressant-like activity than desvenlafaxine.

## Discussion

The primary objective of this study was to investigate the effects of LPM570065, a novel TRI, on 5-HT, DA and NE neurotransmission in the rat striatum. Using microdialysis, the HPLC detection method and the forced swim test during acute and chronic administration, we have demonstrated three primary findings: a) compared with desvenlafaxine, LPM570065 exhibited greater efficacy on enhancing 5-HT, NE and DA neurotransmission and induced more reduction of the immobility time in the forced swim test; b) LPM570065 attenuated the harmful effects of the excessive activation of inhibitory 5-HT_1A_ autoreceptors induced by desvenlafaxine; and c) in contrast to WAY-100635+desvenlafaxine, WAY-100635+LPM570065 showed a capped increase in extracellular 5-HT levels. Overall, these data suggest that this new TRI may be a promising candidate medication to treat depression because it affects both the monoamine system, which is currently targeted by SSRIs and SNRIs, and dopamine reuptake.

SSRIs and SNRIs, which increase extracellular 5-HT and/or NE levels by inhibiting monoamine reuptake in the synaptic cleft, are currently used antidepressants and are still the standard of care at most places. These agents are often effective; however, it takes several weeks to produce the antidepressant effect in patients taking SSRIs or SNRIs. The insufficient effect has been postulated as resulting from inhibitory 5-HT_1A_ autoreceptor activation in the somatodendritic cell body region of the dorsal raphe nucleus [29], [37], [38]. Acute treatment with SSRIs or SNRIs directly blocks 5-HT transporters and causes an increase in terminal 5-HT concentrations. The increase in 5-HT levels activates 5-HT_1A_ autoreceptors and leads to inhibited firing rates in serotonergic neurons. After chronic treatment with SSRIs or SNRIs, 5-HT_1A_ autoreceptors desensitise gradually. The inhibitory feedback mediated by 5-HT_1A_ autoreceptors is coincidentally weakened, which results in increased extracellular 5-HT concentrations [37], [39], [40]. Pre-treatment with WAY-100635 (a 5-HT_1A_ antagonist) may antagonise tonic self-inhibition caused by the activity of 5-HT_1A_ autoreceptors, thus leading to increased 5-HT release in the presence of SSRIs or SNRIs and most likely imitating their chronic effects [29], [41]. Desvenlafaxine is an SNRI and has been used in clinical applications. Our present study showed that acute treatment with desvenlafaxine did not acutely elevate the extracellular 5-HT levels in the rat striatum, which was consistent with previous studies conducted by Alfinito et al. [29], [30] and the hypothesis mentioned above. Although chronic administration of desvenlafaxine induced a greater increase in extracellular 5-HT levels compared with acute administration, the elevation of 5-HT induced by chronic administration of desvenlafaxine for 14 days was still lower than the levels induced by acute administration of desvenlafaxine in combination with WAY-100635. An alternative explanation is that treatment with desvenlafaxine for 14 days led to partial desensitisation of 5-HT_1A_ autoreceptors rather than complete receptor desensitisation. Both previous and present studies suggest that SSRIs and SNRIs suffer from insufficient theory and demonstrate multiple limits in therapeutic efficacy.

As a carboxylic acid ester prodrug of desvenlafaxine, LPM570065 is slightly soluble in water and exhibits enhanced lipophilicity compared with desvenlafaxine (Yunyun Gai, unpublished results). These physical properties provide possibilities for LPM570065 to overcome various barriers *in vivo* to drug delivery. Our present study showed the consistent result that LPM570065 penetrated the rat striatum more rapidly and then exhibited larger total exposure compared with desvenlafaxine. Once in the body, LPM570065 is metabolised to its active parent drug (desvenlafaxine) by ubiquitous esterases [42]; therefore, two pharmacologically active agents, LPM570065 and desvenlafaxine, coexisted *in vivo* for a period of time. As a TRI, LPM570065 enhanced DA, 5-HT and NE neurotransmission compared with desvenlafaxine, which only enhanced 5-HT and NE neurotransmission. Although LPM570065 exhibited several of the insufficient characteristics desvenlafaxine had on elevating extracellular 5-HT levels in the rat striatum, chronic administration of LPM570065 oral suspension actually induced greater efficacy in increasing extracellular 5-HT levels compared with the relative administration of desvenlafaxine. The present study found that oral suspension and intravenous solution, but not oral solution, of LPM570065 markedly increased DA levels. The reason for this finding might be as follows: The total exposure of LPM570065 (expressed as AUC_0–240 min_ values) after treatment with LPM570065 was greatest using intravenous solution, followed by oral suspension and oral solution as the administration routes, and the increases in DA levels observed when LPM570065 was administered via different administration routes were consistent with the exposure of LPM570065 obtained with these different administration routes. Additionally, LPM570065 did not robustly inhibit DA reuptake *in vitro* (IC_50_∶491 nM). Hence, the exposure of LPM570065 administered by oral solution might not be sufficient to induce the marked increases in DA levels observed with LPM570065 administration by oral suspension and intravenous solution. We also used a classical behavioural screening approach (the forced swim test) to evaluate the antidepressant-like activity of LPM570065 relative to desvenlafaxine. The forced swim test in rats showed that acute and chronic administration of LPM570065 reduced the immobility time more than the relative administration of desvenlafaxine. Although the test is not the model of depression *per se*, it is widely used and demonstrates a high specificity for evaluating novel antidepressant drugs [17], [43], [44]. In addition, LPM570065 at the oral dose of 0.06 mmol·kg^−1^ did not increase locomotor activity (Renyu Zhang, unpublished results). Previous studies have demonstrated that the dopamine uptake inhibitors (bupropion 10 mg/kg; nomifensine 2.5 mg/kg), which enhance extracellular DA levels by inhibiting DA reuptake, can reduce the immobility time in the forced swim test without significantly altering locomotor activity [45]. Based on these findings, we speculate that the DA levels increased by acute and chronic administration of LPM570065 might contribute to the improvement of immobility. Of course, further research must be performed to confirm this speculation. All of the above results suggest that LPM570065, as a TRI and a prodrug, may have greater efficacy and/or a more rapid onset of antidepressant action compared with desvelafaxine. This finding has been supported by several reports of combined treatment with methylphenidate and citalopram to accelerate the SSRI response in refractory patients [46], [47]. Furthermore, enhancing dopaminergic neurotransmission improves several comorbid symptoms of depression, such as the experience of pleasure and cognition, the increase in motivated behaviour and the execution of movement [5], [48]. Therefore, this new class of TRIs, such as LPM570065, suggests a promising avenue for improving the insufficient therapeutic effects of current antidepressants.

Besides accelerating the onset of action on enhancing 5-HT neurotransmission, enhancing DA neurotransmission also counterbalanced the effects on 5-HT_1A_ autoreceptors. Our study showed that the extracellular 5-HT concentration was heavily decreased and nearly depleted at 90 min after acute intravenous administration of desvenlafaxine. The finding indicates that desvenlafaxine might excessively activate inhibitory 5-HT_1A_ autoreceptors by inhibiting 5-HT reuptake, which might then lead to excessively inhibition of the firing rates of 5-HT neurons [29], [37], [49], [50]. Although acute intravenous administration of LPM570065 produced larger amounts of desvenlafaxine in the rat striatum compared with the intravenous administration of desvenlafaxine, LPM570065 did not induce such large reductions in extracellular 5-HT levels. Compared with desvenlafaxine, the intravenous administration of LPM570065 increased extracellular DA levels through the inhibition of DA reuptake. Previous studies have reported that DA might activate the serotonergic system through the activation of postsynaptic D_2_-like receptors [51], [52] and the systemic administration of apomorphine elicits increased serotonergic firing rates and increased 5-HT levels [19], [53]. Administration of a D_2_ receptor antagonist (raclopride) attenuated the LPM570065-induced increase in extracellular 5-HT levels within the first 120 min after intravenous administration of LPM570065, and that the reduction in 5-HT levels by raclopride+LPM570065 (i.v.) were less than the extent by desvenlafaxine (i.v.). Both previous studies and the present study have indicated that the desvenlafaxine-induced decrease in 5-HT levels can be attributed to the activation of inhibitory 5-HT_1A_ autoreceptors [29], [37], [49], [50]. Presumably, the elevation of extracellular DA levels might activate D_2_ receptors and then indirectly attenuate the excessive effect of the activation of inhibitory 5-HT_1A_ autoreceptors. In addition to D_2_ receptors, other mechanism might also contribute to attenuating the effect of the activation of inhibitory 5-HT_1A_ autoreceptors. However, the tonic inhibitory feedback of 5-HT_1A_ autoreceptors appears to mask the excitatory effect of LPM570065 on serotonergic neurons. Based on *in vitro* results, this effect is likely because LPM570065, together with its active metabolite desvenlafaxine, has a stronger inhibitory effect on 5-HT reuptake compared with the inhibition of DA reuptake. As a result, the acute intravenous administration of LPM570065 attenuated the reductions in the extracellular 5-HT levels induced by desvenlafaxine; however, a marked increase in the 5-HT levels was not elicited. Intravenous administration of desvenlafaxine increased 5-HT levels to the same extent as LPM570065 in the presence of WAY-100635, but the increase in 5-HT levels observed with oral administration of desvenlafaxine was suppressed. The explanation for this finding might be as follows: a) the desvenlafaxine AUC_0–240 min_ values after oral administrations of desvenlafaxine were lower than that after intravenous administration of desvenlafaxine and were also lower than the cumulative LPM570065 and desvenlafaxine AUC_0–240 min_ values after oral/intravenous administration of LPM570065; b) the lower exposure (expressed as AUC_0–240 min_ value) of desvenlafaxine when it is administered orally might not be sufficient to induce as marked increases in 5-HT levels as intravenous administration of desvenlafaxine and oral/intravenous administration of LPM570065. Therefore, we speculate that the suppressed increases in 5-HT levels in the oral desvenlafaxine groups might be attributed to the lower exposure of desvenlafaxine. Our study also showed that acute administration of LPM570065 in combination with WAY-100635 exhibited a ceiling effect on increasing the extracellular 5-HT levels; however, WAY-100635+desvenlafaxine induced a fully dose-dependent effect on the 5-HT levels. Compared with desvenlafaxine, LPM570065 exhibited the neurochemical activity on enhancing DA levels. Previous study has found that combined administration of 5-HT, NE and DA reuptake inhibitors exhibited reciprocal effects on the 5-HT and DA levels [54]. A possible explanation could be that the elevation of DA induced by LPM570065 appears to have a reciprocal action on the enhancement in the extracellular 5-HT levels. Overall, these data indicate that DA indirectly produces balancing effects on 5-HT_1A_ autoreceptors to maintain normal 5-HT neurotransmission function. These balancing effects enable TRIs to have the potential to reduce 5-HT_1A_ receptor-related adverse events produced by current antidepressants (SNRIs like desvenlafaxine).

Preclinical and preliminary clinical findings demonstrate that amitifadine (EB-1010, formerly DOV 21,947), a novel TRI that increases extracellular 5-HT, DA and NE levels by inhibiting 5-HT, DA and NE transporters, exhibits significant antidepressant efficacy and a benign adverse event profile which is comparable to placebo [55]–[58]. The IC_50_ values of amitifadine inhibition of 5-HT, DA and NE reuptake *in vitro* are 76, 204 and 283 nM, respectively [17]. This finding provides strong positive support that the TRIs that are being developed as a new class of antidepressant may have better efficacy and fewer adverse effects compared with currently used antidepressants.

These findings are encouraging; however, several limitations to the currently study should be considered as we move forward with investigating the potential clinical utility of TRIs such as LPM57006. We chose one brain region (the striatum) in which to study the neurochemical activity of LPM570065 and desvenlafaxine in the present study; other brain regions, such as the frontal cortex, hippocampus and hypothalamus, which are involved in depression and monoamine interactions, will be considered in our future studies. In addition, several behavioural tests and animal models will be introduced to confirm the potential antidepressant effect, safety, side effect profile and feasibility of LPM570065 compared with desvenlafaxine.

Although significant work is necessary to develop LPM570065 as a new antidepressant, preliminary results in the present study still suggests that LPM570065 is a promising candidate medication. Major depression is a common and heterogeneous nervous system disorder triggered by a complex pattern of genetic, epigenetic, developmental, and environmental factors [59]; therefore, it is extremely inappropriate to focus on a single target to develop antidepressants. Agents that interact with several complementary targets or with distributed cerebral networks offer greater hope for developing attractive pharmacotherapeutic approaches [60]. TRIs such as LPM570065 offer a multitarget concept for developing antidepressants. Following this conceptual base, multiple efficacious antidepressants can be developed in the near future.

## Conclusion

As a new TRI and a desvenlafaxine prodrug, LPM570065 exhibited greater efficacy in increasing extracellular 5-HT, DA and NE levels and better antidepressant-like activity in the forced swim test after acute and chronic administration compared with desvenlafaxine. In addition, LPM570065 counterbalanced the harmful effects of desvenlafaxine on 5-HT neurotransmission (related to 5-HT_1A_ autoreceptors) due to the neurochemical activity of LPM570065 on extracellular DA levels. These results offer great hope for TRIs to exhibit efficacious treatment of both the cardinal and comorbid symptoms of depression. This study also indicates that this new class of putative antidepressants (i.e., TRIs such as LPM570065) may have the potential to reduce 5-HT_1A_ receptor-related adverse events relative to SNRIs (and certainly relative to the SSRIs that are still the standard of care). TRIs such as LPM570065 offer a multitarget concept to develop efficacious antidepressants and have the potential to provide a new therapeutic mechanism. Furthermore, these findings may also challenge the dogma that a prodrug with activity is a disadvantage for drug development. To further confirm the antidepressant effect of LPM570065, our future studies will focus on microdialysis studies in other brain regions and will utilize several behavioural tests in animal models of depression.
